# A Novel Underdetermined Blind Source Separation Method and Its Application to Source Contribution Quantitative Estimation

**DOI:** 10.3390/s19061413

**Published:** 2019-03-22

**Authors:** Jiantao Lu, Wei Cheng, Yanyang Zi

**Affiliations:** State Key Laboratory for Manufacturing Systems Engineering, Xi’an Jiaotong University, Xi’an 710049, China; lujiantao1990@stu.xjtu.edu.cn (J.L.); ziyy@xjtu.edu.cn (Y.Z.)

**Keywords:** underdetermined blind source separation, source contribution estimation, single source point, mixing matrix estimation

## Abstract

To identify the major vibration and radiation noise, a source contribution quantitative estimation method is proposed based on underdetermined blind source separation. First, the single source points (SSPs) are identified by directly searching the identical normalized time-frequency vectors of mixed signals, which can improve the efficiency and accuracy in identifying SSPs. Then, the mixing matrix is obtained by hierarchical clustering, and source signals can also be recovered by the least square method. Second, the optimal combination coefficients between source signals and mixed signals can be calculated based on minimum redundant error energy. Therefore, mixed signals can be optimally linearly combined by source signals via the coefficients. Third, the energy elimination method is used to quantitatively estimate source contributions. Finally, the effectiveness of the proposed method is verified via numerical case studies and experiments with a cylindrical structure, and the results show that source signals can be effectively recovered, and source contributions can be quantitatively estimated by the proposed method.

## 1. Introduction

Vibration and radiation noise have a significant effect on the safety and stability of some mechanical systems [1,2], for example, excessive noise of underwater vehicle will interfere with its own detection accuracy. Independently acquiring information from each source of the mechanical system can help to quickly judge its running state. However, in practice, the information measured by sensors is the superposition of some sources, because different components of the mechanical system will interfere with each other, which makes it difficult to directly measure the source information [3]. Therefore, some supplementary signal processing methods are needed to further process the collected information to obtain the expected source signals [1]. Among post-processing approaches, blind source separation (BSS) has demonstrated its usefulness in separating sources from mixed signals, due to its simplicity and effectiveness. More importantly, BSS can be utilized without the structure models and the transmission paths that are difficult to be obtained, and therefore BSS has been widely used in practice [4,5,6,7,8,9]. However, most of these methods are mainly designed for (over)determined BSS where the number of sensors is no smaller than that of sources, and thus they may fail when dealing with underdetermined cases. Therefore, we mainly address the problem of underdetermined BSS (UBSS) where the number of mixed signals is smaller than that of sources in this study. In addition, reducing the vibration of the major sources rather than all sources can achieve satisfactory results with smaller cost [1,10]. Therefore, how to evaluate source contribution quantitatively is another problem to be addressed in our study.

Recently, UBSS has gained considerable research interests and various excellent methods have been proposed to tackle this problem. The sparsity of source signals is often exploited for UBSS. Bofill and Zibulevsky proposed a two-step algorithm, i.e., mixing matrix estimation and source recovery, that exploits the sparsity of source signals in the frequency domain [11]. The framework of the two-step algorithm is used by many researchers later. Wigner-Ville distribution (WVD) is usually adopted to transform mixed signals from the time domain to time-frequency (TF) domain, which can greatly increase the sparsity of source signals. Aïssa-El-Bey et al. proposed an effective WVD-based UBSS method that does not require disjoint source signals in the whole TF domain and only needs some TF points where only one source exists, called single source points (SSPs) [12]. After obtaining the mixing matrix, source signals are recovered by the subspace method under the assumption that the number of active source signals must be smaller than sensors at any TF point. Then, this restriction is relaxed by Peng and Xiang, which only requires that the number of active source signals at any TF point should not exceed that of sensors [13]. Xie et al. further relaxed the sparsity constraint and developed a new WVD-based UBSS algorithm to recover the sources exactly at every auto-term TF point, provided that the number of the sources is smaller than the twice of the sensors [14]. Though WVD can provide a high concentration of signal energy in the TF domain, it has some inherent disadvantages of cross-term disturbance and high computational complexity. Therefore, short-time Fourier transform (STFT) is also adopted by many researchers recently [15,16], due to its freedom of cross terms and its easiness to be inverted. Reju et al. proposed a mixing matrix estimation algorithm based on STFT and proved that a TF point is an SSP when the absolute direction of the real and the imaginary parts of TF vectors of the mixed signals are the same [17]. Li et al. also proposed an effective algorithm to identify SSPs by utilizing time-frequency coefficients of the mixed signals and the complex conjugates of the coefficients [18]. However, most of the SSPs-based UBSS methods focus merely on the nature of single SSP ignoring the relationship between SSPs, which leads to low identification accuracy of SSPs, especially in noisy cases. Zhen et al. proposed a UBSS method based on STFT in which SSPs are identified by sparse coding. Since the sparse coding strategy considers the linear relations between SSPs, Zhen’s method can obtain excellent estimation performance even in low signal to noise ratio (SNR) cases [19]. However, the SSPs identification effectiveness of Zhen’s method is still low when SSPs are rare. Furthermore, it is a great challenge to select the regularization parameter, since improper parameter will lead to larger errors of the estimated sources. Hence, it is still difficult to identify SSPs accurately and efficiently.

After source signals are recovered, source contributions can be quantitatively estimated. According to the percentage of the source contribution, the major sources to the measurement points can also be identified. Cheng et al. proposed a source contribution evaluation method of mechanical vibration signals via enhanced independent component analysis for complete BSS [4,11]. Zhang et al. proposed a source contribution method with kurtosis-based constrained independent component analysis, which can quantitatively estimate source contribution by reducing the energy of the extracted independent components from mixed signals in each extraction [1]. However, the separation matrix should be an orthogonal matrix, which cannot be satisfied in UBSS. In this paper, we are mainly addressing the issue of source contribution estimation for UBSS.

To accurately recover source signals and identify the major sources, an effective UBSS method and a source contribution quantitative estimation method are proposed, in which SSPs can be identified by directly searching the identical normalized TF vectors of mixed signals instead of the sparse coding in Zhen’s method. After source signals are well recovered by the proposed UBSS method, the optimal combination coefficients between source signals and mixed signals can be obtained based on minimum redundant error energy. Then, the contributions of the recovered source signals are quantitatively estimated by using energy elimination method. Finally, the effectiveness of the proposed method is verified by some numerical simulations and experimental studies on a cylindrical shell structure.

The organization of this paper is as follows. In Section 2, an effective UBSS method is proposed. In Section 3, the source contribution quantitative estimation method is given. In Section 4, the proposed UBSS method and the contribution quantitative estimation method are verified by the numerical case studies. In Section 5, the proposed method is further verified with experimental studies on a cylindrical shell structure. Some conclusions are drawn in Section 6.

## 2. Underdetermined Blind Source Separation

### 2.1. Basic Theory

The linear instantaneous mixed model of UBSS can be expressed as

(1) x(t)=As(t),

where $x(t)={\left[x_{1}(t),\;x_{2}(t),\;\ldots ,x_{N}(t)\right]}^{T}$ and $s(t)={\left[s_{1}(t),\;s_{2}(t),\;\ldots ,s_{M}(t)\right]}^{T}$ are the mixed vector and the source vector in the time domain, respectively, and $\cdot^{T}$ represents the transpose operation; *N* and *M* (*N* < *M*) are the number of mixed signals and source signals, respectively; $A=[a_{1},\;a_{2},\;\ldots ,\;a_{M}]$ is the mixing matrix with $a_{i}$ as its column. The aim of UBSS is to estimate source signals without any prior information of s(t) or A, except that *N* < *M*.

To increase the sparsity of source signals, the above linear instantaneous mixed model can be transformed into the TF domain as Equation (2) or Equation (3) by STFT.

(2) X(t,f)=AS(t,f),

(3) $$X\left(t,f\right)=\sum_{i=1}^{M}a_{i}S_{i}\left(t,f\right),$$

where $X\left(t,f\right)={\left[X_{1}\left(t,f\right),\;X_{2}\left(t,f\right),\;\ldots ,X_{N}\left(t,f\right)\right]}^{T}$ and $S\left(t,f\right)={\left[S_{1}\left(t,f\right),\;S_{2}\left(t,f\right),\ldots ,S_{M}\left(t,f\right)\right]}^{T}$ are the STFT coefficients of x(t) and s(t) at TF point (t,f), respectively.

### 2.2. Proposed Mixing Matrix Estimation Method

The ideal goal of UBSS is to estimate source signals without any prior information, except that N<M. Actually, it seems almost impossible to obtain an effective estimation of source signals if we know nothing about s(t) or A. Therefore, some assumptions are given first.

**Assumption** **1.** *For each source signal $s_{i}(t)$, there are some TF points (t,f) where only $S_{i}(t,f)$ is dominant, i.e., $\left|S_{i}(t,f)\right|\gg \left|S_{j}(t,f)\right|$, ∀ j≠i.*

**Assumption** **2.** *Source signals are mutually independent.*

**Assumption** **3.** *Any N×N submatrix of the mixing matrix A is of full rank.*

These three assumptions could hold in many practical cases and have been widely used in recent UBSS methods. Assumption 1 is used to guarantee the existence of SSPs in the process of mixing matrix estimation. However, Assumption 1 is not necessary for recovering source signals, that is, if the mixing matrix is known or can be estimated by other methods, Assumption 1 can be removed. Assumption 2 and Assumption 3 are used to increase the stability of the SSPs identification method. Assumption 2 is also used to guarantee that there is no cross energy among source signals, which will be used in the source contribution estimation. Besides, Assumption 3 is also used to guarantee that all source signals could be correctly recovered.

Now, at any TF point, say (u,v), if only one source is active, say $S_{i}(u,v)$, i.e., (u,v) is an SSP corresponding to $s_{i}(t)$, then Equation (3) can be rewritten as

(4) $$X(u,v)=S_{i}(u,v)a_{i}.$$

Equation (4) shows that the TF vector of mixed signals at TF point (u,v) is collinear with the i-th column of the mixing matrix. It can be also obtained that all TF vectors of mixed signals at SSPs corresponding to $s_{i}(t)$ will be collinear with $a_{i}$, that is, all SSPs corresponding to the same active source could be linearly represented by each other. Assume that $S_{i}\left(\psi ,\omega \right)$ is also an SSP corresponding to $s_{i}(t)$, then we will obtain

(5) X(u,v)=rX(ψ,ω),

where r is a real coefficient.

Now, the problem is how to identify the TF vectors that satisfy Equation (5) from all TF vectors of mixed signals.

From Equation (4), X(u,v) can be normalized as

(6) $$\begin{array}{ll} \tilde{X}\left(u,v\right) & =\frac{S_{i}\left(u,v\right)a_{i}}{{\left\|S_{i}\left(u,v\right)a_{i}\right\|}_{2}}\\ & =\frac{a_{i}}{{\left\|a_{i}\right\|}_{2}}\\ & ={\tilde{a}}_{i} \end{array},$$

where ῀ represents the normalized vector and ${\left\|\right\|}_{2}$ is the 2-norm. Similarly, the normalized vector of X(ψ,ω) can also be written as

(7) $$\tilde{X}\left(\psi ,\omega \right)={\tilde{a}}_{i}$$

As shown in Equations (6) and (7), all the normalized TF vectors at the SSPs corresponding to the *i*-th dominant source will be equal to the normalized vector of $a_{i}$. Therefore, SSPs can be identified by searching the identical normalized TF vectors of mixed signals, i.e.,

(8) $$\tilde{X}(u,v)=\tilde{X}(\psi ,\omega )$$

Equations (5) and (8) are equivalent. Therefore, SSPs can be identified by checking whether normalized TF vectors are identical or not. As all vectors have been normalized, they will be identical if the directions of vectors are the same. The cosine of the angle between $\tilde{X}\left(u,v\right)$ and $\tilde{X}\left(\psi ,\omega \right)$ can be calculated by

(9) $$\begin{array}{ll} \cos\left[\tilde{X}\left(u,v\right),\;\tilde{X}\left(\psi ,\omega \right)\right] & =\frac{{\tilde{X}}^{T}\left(u,v\right)\;\tilde{X}\left(\psi ,\omega \right)}{{\left\|\tilde{X}\left(u,v\right)\right\|}_{2}{\left\|\tilde{X}\left(\psi ,\omega \right)\right\|}_{2}}\\ & =\langle \tilde{X}\left(u,v\right),\;\tilde{X}\left(\psi ,\omega \right)\rangle \end{array},$$

where $\langle \tilde{X}\left(u,v\right),\;\tilde{X}\left(\psi ,\omega \right)\rangle$ is the scalar product of $\tilde{X}\left(u,v\right)$ and $\tilde{X}\left(\psi ,\omega \right)$.

Therefore, Equation (8) will hold if

(10) $$1-\langle \tilde{X}\left(u,v\right),\;\tilde{X}\left(\psi ,\omega \right)\rangle =0$$

Noise effect is not considered in the above derivation. In noisy environments, we cannot find SSPs that exactly satisfies Equation (10). Instead, we can get SSPs from the following criterion:

(11) $$\left|1-\langle \tilde{X}\left(u,v\right),\;\tilde{X}\left(\psi ,\omega \right)\rangle \right|<\delta_{1},$$

where |·| is the absolute value of ·, and $\delta_{1}$ is the SSPs threshold close to zero. Therefore, both $\tilde{X}\left(u,v\right)$ and $\tilde{X}\left(\psi ,\omega \right)$ are regarded as SSPs if they satisfy Equation (11).

As stated in [20], most of the signal energy will be concentrated in nearly 10% of the frequency bins. Therefore, in our study, the frequency bins are sorted in descending order according to their variances and only $N_{f}$ frequency bins with larger variance are selected for identifying SSPs. Moreover, we recommend that the data could be segmented when the sampling length is very large, and the results obtained in different segments can be combined by the similarity of the signal itself.

SSPs threshold $\delta_{1}$ has a large effect on the accuracy of SSPs and we now discuss how to choose the SSPs threshold. If $\delta_{1}$ is too small, the accuracy of SSPs will increase, however, the number of identified SSPs each time will decrease so that the efficiency of identifying enough SSPs will decline. Too small thresholds will even lead to insufficient SSPs. Otherwise, if $\delta_{1}$ is too large, the criterion becomes loose and too many outliers will be misjudged as SSPs, which will reduce the accuracy of mixing matrix estimation and source recovery. Since $\delta_{1}$ is related to the property of source signals, it is hard to give a unified range for all kinds of signals. A feasible approach that considers both efficiency and accuracy is to set a smaller threshold $\delta_{1}$ and a minimum number $N_{\mathrm{min-SSPs}}$ of identified SSPs. If the number $N_{\mathrm{SSPs}}$ of extracted SSPs is smaller than $N_{\mathrm{min-SSPs}}$, the threshold will be doubled. When source signals contain large noise or source signals are not very sparse in the TF domain, the threshold will gradually increase, which can reduce the effect of using an unsuitable threshold.

In general, TF vectors with negligible energy are greatly influenced by noise, which will easily lead to the misjudgment of SSPs. To obtain effective SSPs, these vectors should be removed before identifying SSPs if they satisfy

(12) $${\left\|X\left(u_{0},v_{0}\right)\right\|}_{2}<\delta_{2}\bar{{\left\|X\right\|}_{2}},$$

where $\delta_{2}$ is a threshold close to zero and $\bar{{\left\|X\right\|}_{2}}$ represents the average of 2-norm of all TF vectors.

After identifying SSPs, the next stage is to estimate the mixing matrix. It can be found from Equations (6) and (7) that the identified SSPs are the set of normalized column vectors of the mixing matrix. Therefore, the mixing matrix can be estimated by clustering these TF vectors and the hierarchical clustering technique [21,22] is used here. It should be noted that this clustering technique may not be the best algorithm to cluster SSPs as other algorithms can also be used [23]. More details on adjusting the cluster number can be found in [17]. As studied in [17], the mixing matrix estimation error can be further reduced by removing the points which are away from the mean direction of the cluster. This strategy is also used in our study and the outlier detection rule is the same as [17]. By re-clustering SSPs after elimination of the outliers, each column of $\hat{A}$ can be obtained via calculating the center of each cluster.

### 2.3. Source Recovery

**Assumption** **4.** *At each TF point, the number of source signals is smaller than that of mixed signals.*

Even though $\hat{A}$ is known, the solution of the system in Equation (1) is not unique. Actually, source signals can be recovered by a series of least square problems [19] with Assumption 3 and Assumption 4, which minimizes the error function by selecting the optimal N×(N−1) submatrix of $\hat{A}$. Let A be a set composed of all N×(N−1) submatrices of $\hat{A}$, that is

(13) $$A=\left\{A_{\;i}|A_{\;i}=\left[{\hat{a}}_{\theta_{1}},{\hat{a}}_{\theta_{2}},\ldots ,{\hat{a}}_{\theta_{N-1}}\right]\right\}$$

Then for each TF point (t, k), there exists $A_{\;*}=\left[{\hat{a}}_{\phi_{1}},{\hat{a}}_{\phi_{2}},\ldots ,{\hat{a}}_{\phi_{N-1}}\right]$ that satisfies

(14) $$X\left(t,\;k\right)=A_{\;*}A_{\;*}^{†}X\left(t,\;k\right),$$

where ${}^{†}$ is the pseudo-inverse of a matrix. Then, source signals can be estimated by

(15) $${\hat{S}}_{j}\left(t,\;k\right)=\{\begin{array}{ll} \begin{array}{l} e_{i},\\ 0, \end{array} & \begin{array}{l} \mathrm{if}\;j=\phi_{i}\\ \mathrm{otherwise} \end{array}, \end{array}$$

where $e={\left[e_{1},\;e_{2},\;\ldots ,\;e_{N-1}\right]}^{T}=A_{\;*}^{†}X\left(t,\;k\right)$, and $A_{\;*}$ can be obtained by

(16) $$A_{\;*}=\arg\;\underset{A_{\;i}\in A}{\min}{\left\|X\left(t,\;k\right)-A_{\;i}A_{\;i}^{†}X\left(t,\;k\right)\right\|}_{2}$$

Finally, the time domain of the estimated source signals $\hat{S}\left(t\right)$ can be easily obtained by inverse STFT.

## 3. Proposed Source Contribution Estimation Method

Unlike the determined BSS, mixed signals in UBSS usually cannot be linearly represented by the estimated source signals, due to noise and separation errors, i.e., there exists a residual between mixed signals and the estimated source signals. Therefore, the *i*-th mixed signal $x_{i}$ can be expressed as

(17) $$x_{i}=w_{i}^{T}\hat{S}+z_{i},$$

where $w_{i}={\left[w_{i1},\;w_{i2},\;\ldots ,\;w_{iM}\right]}^{T}$ represents the coefficients and $z_{i}$ represents the residual. It should be noted that $x_{i}={\left[x_{i}\left(1\right),x_{i}\left(2\right),\cdots ,x_{i}\left(T\right)\right]}_{1\times T}$ represents the whole discrete sequence of the *i*-th mixed signal and $\hat{S}={\left[s_{1}^{T},s_{2}^{T},\ldots ,s_{M}^{T}\right]}^{T}$ is also the whole discrete sequences of *M* estimated source signals, that is, $\hat{S}$ is a M×T matrix. $z_{i}={\left[z_{i}\left(1\right),z_{i}\left(2\right),\cdots ,z_{i}\left(T\right)\right]}_{1\times T}$ represents the *i*-th residual signal that has the same dimension as $x_{i}$. Then, a problem arises that how much $\hat{S}$ is contained in $x_{i}$. This problem can be addressed based on minimum redundant error energy, i.e.,

(18) $$\begin{array}{ll} \underset{w_{i}}{\min} & {\left\|z_{i}\right\|}_{2}^{2} \end{array}$$

Therefore, the problem can be transformed into how to find the optimal coefficients in Equation (18).

Let $f\left(w_{i}\right)={\left\|z_{i}\right\|}_{2}^{2}$, it is easy to see that $f\left(w_{i}\right)$ is a continuous differential function. From Equation (17), we can obtain

(19) $$\begin{array}{ll} f\left(w_{i}\right) & ={\left\|x_{i}-w_{i}^{T}\hat{S}\right\|}_{2}^{2}\\ & =\left[x_{i}-w_{i}^{T}\hat{S}\right]{\left[x_{i}-w_{i}^{T}\hat{S}\right]}^{T}\\ & =x_{i}x_{i}^{T}-x_{i}{\hat{S}}^{T}w_{i}-w_{i}^{T}\hat{S}x_{i}^{T}+w_{i}^{T}\hat{S}{\hat{S}}^{T}w_{i} \end{array}$$

The derivative of $f\left(w_{i}\right)$ with respect to $w_{i}$ can be calculated by

(20) $$\nabla_{w_{i}}\left[f\left(w_{i}\right)\right]=2\left[\hat{S}{\hat{S}}^{T}w_{i}-\hat{S}x_{i}^{T}\right]$$

Let $\nabla_{w_{i}}\left[f\left(w_{i}\right)\right]=0$, we will have

(21) $$w_{i}={\left(\hat{S}{\hat{S}}^{T}\right)}^{-1}\hat{S}x_{i}^{T}$$

From Assumption 2, source signals are mutually independent, therefore, the estimated source signals are also approximately mutually independent, that is, the rank of $\hat{S}$ is *M*. Therefore, the rank of $\hat{S}{\hat{S}}^{T}$ is also *M*, that is, $\hat{S}{\hat{S}}^{T}$ is a matrix with full rank. Thus, $f\left(w_{i}\right)$ has only one stationary point. The Hessian matrix of $f\left(w_{i}\right)$ is

(22) $$\nabla_{w_{i}}^{2}f\left(w_{i}\right)=2\hat{S}{\hat{S}}^{T}$$

The Hessian matrix of $f\left(w_{i}\right)$ is nonnegative definite. Therefore, from Equations (21) and (22), the minimum value of $f\left(w_{i}\right)$ can be obtained at $w_{i*}={\left(\hat{S}{\hat{S}}^{T}\right)}^{-1}\hat{S}x_{i}^{T}$.

Based on the above analysis, the optimal combination coefficients of $\hat{S}$ to all mixed signals can be obtained. Then, source contributions can be quantitatively estimated using $w_{*}$. The following equation can be obtained.

(23) $$\begin{array}{llll} x_{i-j}=x_{i}-w_{i*,\;j}{\hat{s}}_{j}, & i=1,2,\ldots ,N & \mathrm{and} & j=1,2,\ldots ,M \end{array},$$

where $x_{i-j}$ represents the vector of $x_{i}$ that subtracts the contribution of ${\hat{s}}_{j}$, and $w_{i*,\;j}$ is the *j*-th element of $w_{i*}$. From Assumption 2, there is no cross energy among source signals, then the contribution $C_{ij}$ of the *j*-th estimated source signals ${\hat{s}}_{j}$ to the *i*-th mixed signals $x_{i}$ are calculated according to

(24) $$C_{ij}=1-\frac{{\left\|x_{i-j}\right\|}_{2}^{2}}{{\left\|x_{i}\right\|}_{2}^{2}}$$

Generally, due to the noise and estimation error, the sum of the contributions of all estimated source signals to a mixed signal is not equal to 1, which is different from complete BSS. From Equation (24), $C_{ij}<0$ if ${\left\|x_{i-j}\right\|}_{2}^{2}>{\left\|x_{i}\right\|}_{2}^{2}$, which implies that the *j*-th estimated source signals can decrease the overall vibration energy.

**Figure 1:**
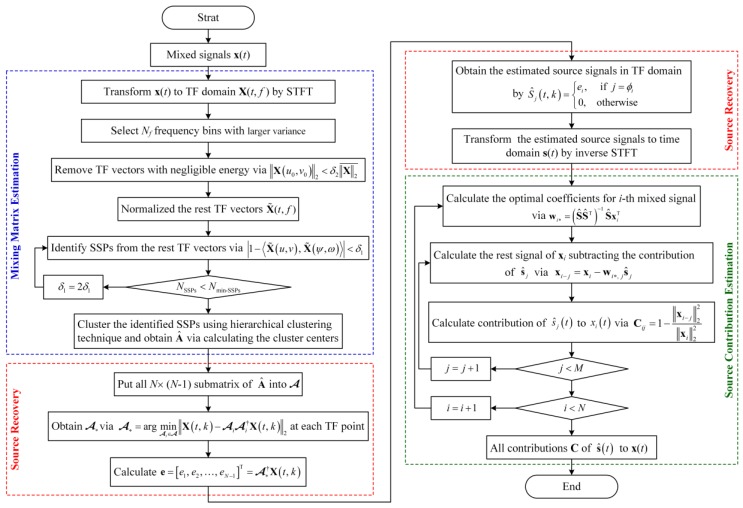
Flowchart of the proposed undetermined blind source separation (UBSS)-based source contribution estimation method.

The flowchart of the proposed UBSS-based source contribution estimation method is shown in Figure 1.

## 4. Numerical Case Study

### 4.1. Performance of the Proposed UBSS Method

In this section, we mainly evaluate the separation performance of the proposed UBSS method with different sample sizes and different numbers of mixed signals. Some numerical case studies are conducted using five artificial source signals: $s_{1}\left(t\right)$ is a low frequency sinusoidal wave; $s_{2}\left(t\right)$ is a high frequency sinusoidal wave; $s_{3}\left(t\right)$ is a periodic wave with amplitude modulation; $s_{4}\left(t\right)$ is a shock attenuation signal wave; $s_{5}\left(t\right)$ is also a periodic wave with amplitude modulation. The generating functions of the source signals are listed as follows:

(25) $$s(t)=\left[\begin{array}{l} s_{1}\left(t\right)\\ s_{2}\left(t\right)\\ s_{3}\left(t\right)\\ s_{4}\left(t\right)\\ s_{5}\left(t\right) \end{array}\right]=\left[\begin{array}{l} \sin(2\pi \cdot 23t)\\ \sin(2\pi \cdot 281t)\\ \sin(2\pi \cdot 10t)\cos(2\pi \cdot 105t)\\ \begin{array}{l} 5\sum_{n=0}^{44}\left\{\begin{array}{l} \sin[2\pi \cdot 995t(t-0.023n)]\\ \exp[-628(t-0.023n)]u(t-0.023n) \end{array}\right\}\\ \sin(2\pi \cdot 10t)\cos(2\pi \cdot 710t) \end{array} \end{array}\right]$$

Two, three and four mixed signals are generated by these five source signals. In each case, the averages of 50 Monte Carlo simulations are used to evaluate the performance of the proposed method, and in each simulation, Gaussian white noise with SNR = 10 dB is independently added to each source signal. The sampling frequency is 10 kHz. In the proposed method, the window length is 1024 and window overlap is 256, the number of selected frequency bins $N_{f}=80$, initial SSPs threshold *δ*_1_ = 0.0001, minimum number of SSPs $N_{\mathrm{min-SSPs}}=300$ and energy threshold $\delta_{2}=0.1$.

To quantitatively verify the better performance of the proposed method, SNRs of $\hat{A}$ and $\hat{s}(t)$ are calculated by Equations (26) and (27), respectively.

(26) $$E_{A}(i)=10\log\left({\left\|{\hat{a}}_{i}\right\|}_{2}^{2}/{\left\|{\hat{a}}_{i}-a_{i}\right\|}_{2}^{2}\right),$$

where $a_{i}$ and ${\hat{a}}_{i}$ are the *i*-th column of A and $\hat{A}$, respectively.

(27) $$E_{s}(i)=10\log\left(\underset{\varsigma }{\min}\;{\left\|{\hat{s}}_{i}(t)\right\|}_{2}^{2}/{\left\|{\hat{s}}_{i}(t)-\varsigma s_{i}(t)\right\|}_{2}^{2}\right),$$

where *ς* is a scalar that reflects the scalar indeterminacies.

**Figure 2:**
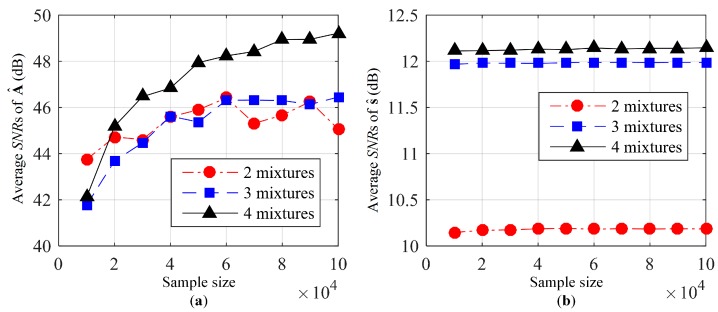
Separation performance with different sample size and different numbers of mixed signals: (**a**) Average signal to noise ratio (SNRs) of estimated mixing matrix; (**b**) average SNRs of estimated source signals.

The average SNRs of the estimated mixing matrix and the estimated source signals are shown in Figure 2a,b, respectively. From Figure 2a, the average SNRs of the estimated mixing matrix will increase with the increase in sample sizes. However, from Figure 2b, the average SNRs of the estimated source signals remained nearly unchanged with the increase in sample size. This is because the average SNRs of the estimated mixing matrix have been more than 40 dB when the sample size is 10,000, which means the mixing matrix is nearly the same as true mixing matrix. It can also be seen from Figure 2 that the separation performance also improves with the increase in the number of mixed signals. Though the average SNRs of the estimated mixing matrix with two mixtures are nearly the same as that with three mixtures, the average SNRs of the estimated source signals differ a lot in these two cases. That is because the number of source signals must be smaller than that of mixed signals at each TF point according to Assumption 4, which means that at most one source exists at each TF point in the case of two mixtures. This restriction is too strict, leading to worse separation performance in source signals.

### 4.2. Performance of the Proposed Source Contribution Estimation Method

In order to validate the effectiveness of the proposed source contribution quantitative estimation method, the following simulations are conducted. Source signals are the first four signals in Equation (25). The mixing matrix are

(28) $$A=\left[\begin{array}{rrrr} 0.5736 & 0.6293 & 0.6561 & 0.7071\\ 0.5587 & -0.5953 & -0.6536 & 0.5930\\ 0.5991 & 0.4995 & -0.3774 & -0.3851 \end{array}\right]$$

The sampling frequency and sampling length is 10 kHz and 1 s, respectively. One hundred Monte Carlo simulations are conducted to evaluate the performance of the proposed method. In each simulation, Gaussian white noise is independently added into each source signal and each mixed signal with SNR = 10 dB and SNR = 15 dB, respectively.

The performance of the proposed UBSS method is compared with Reju’s method [17] and Zhen’s method [19]. Since Reju’s method is designed only for mixing matrix estimation, it cannot recover source signals. Therefore, the mixing matrix estimated by Reju’s method is then inputted into Zhen’s method to estimate source signals. The parameters in different methods are as follows. In all methods, the Hanning window is used in STFT, and the window length is 1024 and window overlap is 256. In Zhen’s method, regularization parameter λ=0.001 and energy threshold $\delta_{2}=0.1$. In Reju’s method, the parameter Δθ is set as $1.5^{\circ }$ and the number of selected frequency bins $N_{f}=80$. The parameters in the proposed method are the same as those in Section 4.1.

**Figure 3:**
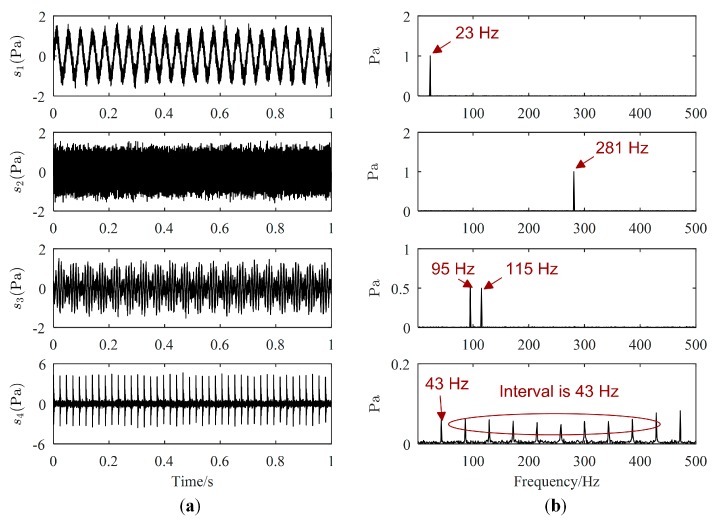
Source signals: (**a**) Waveforms; (**b**) Fourier spectrums.

**Figure 4:**
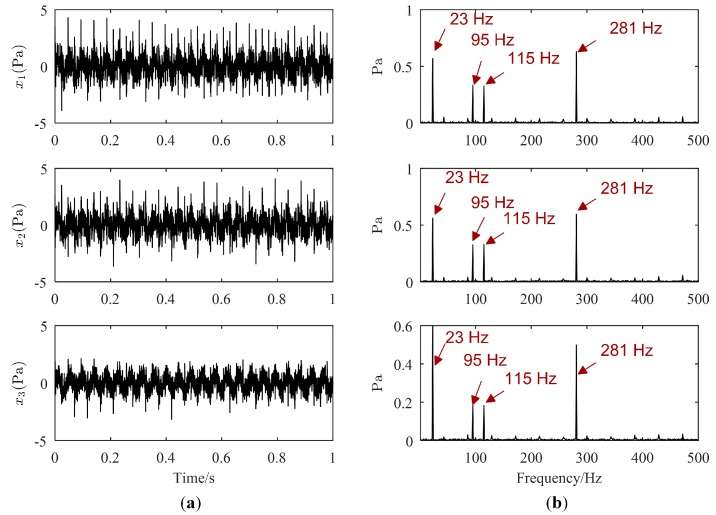
Mixed signals: (**a**) Waveforms; (**b**) Fourier spectrums.

One example of the separation results is as follows. Waveforms and Fourier spectrums of source signals are displayed in Figure 3, while the major frequencies of the source signals can be easily obtained from Figure 3b. The major frequencies of $s_{1}(t)$, $s_{2}(t)$, and $s_{4}(t)$ are 23 Hz, 281 Hz, and 43 Hz, respectively, while the major frequencies of $s_{3}(t)$ are 95 Hz and 115 Hz. Waveforms and Fourier spectrums of mixed signals are shown in Figure 4. From Figure 4a, mixed signals are the superposition of source signals, therefore, we cannot directly obtain the waveforms of source signals. From Figure 4b, the major frequencies of source signals can be found in each Fourier spectrum of mixed signals, and the frequencies of $s_{4}(t)$ is overwhelmed by those of other source signals. Therefore, signal processing methods are needed to estimate all source signals.

The estimated mixing matrix of the proposed method is

(29) $$\hat{A}=\left[\begin{array}{rrrr} 0.5720 & 0.6331 & 0.6586 & 0.6975\\ 0.5558 & -0.5950 & -0.6624 & 0.6032\\ 0.6029 & 0.4944 & -0.3568 & -0.3847 \end{array}\right]$$

The absolute differences between A and $\hat{A}$ are calculated in Equation (30), illustrating that the mixing matrix has been well estimated because each of the absolute differences is very small.

(30) $$\left|A-\hat{A}\right|=\left[\begin{array}{rrrr} 0.0016 & 0.0038 & 0.0025 & 0.0096\\ 0.0028 & 0.0003 & 0.0089 & 0.0102\\ 0.0039 & 0.0051 & 0.0206 & 0.0005 \end{array}\right]$$

**Figure 5:**
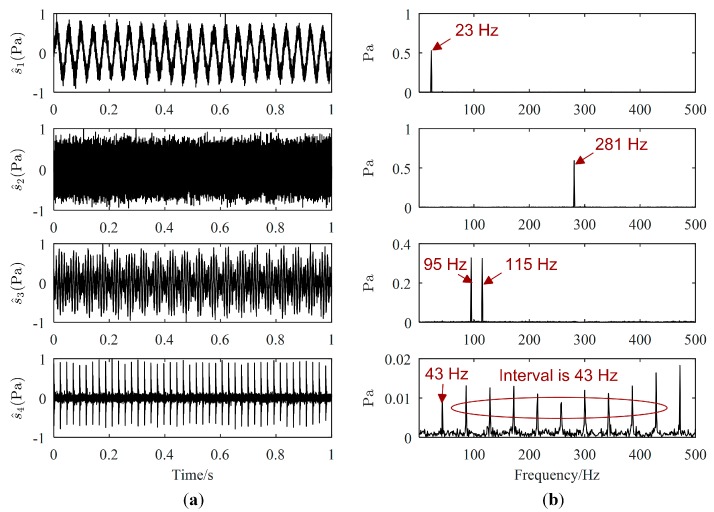
Estimated source signals by the proposed method: (**a**) Waveforms; (**b**) Fourier spectrums.

**Figure 6:**
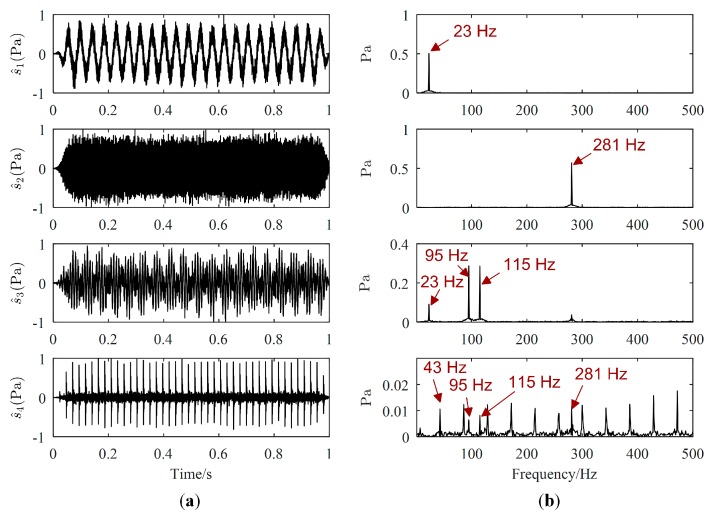
Estimated source signals by Zhen’s method: (**a**) Waveforms; (**b**) Fourier spectrums.

**Figure 7:**
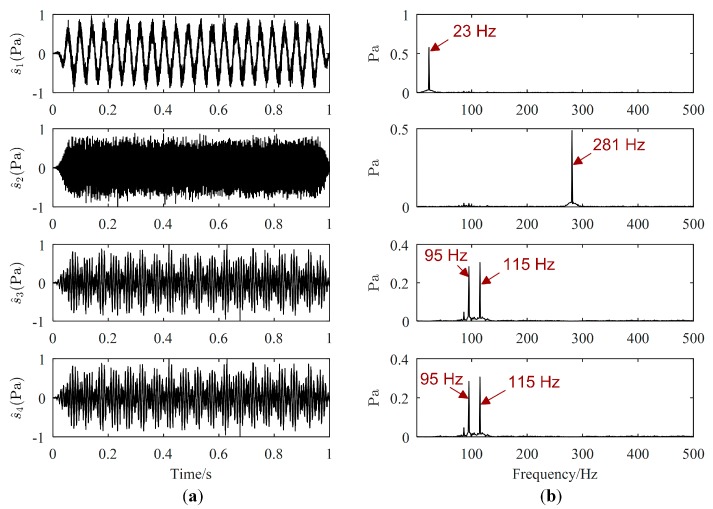
Estimated source signals by Reju’s method: (**a**) Waveforms; (**b**) Fourier spectrums.

Source signals estimated by the proposed method, Zhen’s method and Reju’s method are displayed in Figure 5, Figure 6 and Figure 7, respectively. The order of $\hat{s}(t)$ has been adjusted according to s(t). Comparing Figure 5a with Figure 3a, we could find that the waveforms of $\hat{s}(t)$ are quite similar to those of s(t). From the Fourier spectrums of $\hat{s}(t)$, the major frequencies of s(t) have been well recovered, which can validate the effectiveness of the proposed UBSS method. As revealed by Figure 6a, it seems that the waveforms of s(t) are also well recovered by Zhen’s method. However, as shown in Figure 6b, there is interference frequency 23 Hz in the Fourier spectrums of ${\hat{s}}_{3}(t)$, and interference frequencies 95 Hz, 115 Hz and 281 Hz in the Fourier spectrums of ${\hat{s}}_{4}(t)$, which indicates that source signal ${\hat{s}}_{3}(t)$ and ${\hat{s}}_{4}(t)$ were not well estimated. It could be seen from Figure 7 that $s_{4}(t)$ is not estimated by Reju’s method.

Average SNRs of 100 Monte Carlo simulations of $\hat{A}$ estimated by different methods are listed in Table 1, from which we can see that SNRs of $\hat{A}$ estimated by the proposed method are larger than those estimated by Zhen’s method and Reju’s method. Average SNRs of all columns of the mixing matrix estimated by Zhen’s method, Reju’s method and the proposed method is 18.12 dB, 32.41 dB and 40.65 dB, respectively, which implies that the proposed method could estimate the mixing matrix more accurately. Table 2 shows the average SNRs of 100 Monte Carlo simulations of $\hat{s}\left(t\right)$ estimated by different methods. As can be seen in Table 2, all SNRs of $\hat{s}\left(t\right)$ estimated by the proposed method are also larger than those estimated by Zhen’s method and Reju’s method. Average SNRs of all sources of Zhen’s method, Reju’s method and the proposed method are 8.41 dB, 9.17 dB and 11.66 dB, that is, the average SNR increments of all sources estimated by the proposed method are 38.72% and 27.18% when compared with Zhen’s method and Reju’s method, respectively. The above results tend to validate that the proposed UBSS method performs more effectively than Zhen’s method and Reju’s method.

The running time is used to evaluate the efficiency of the methods. CPU of the computer is Inter Core i5-4590 of 3.30 GHz and RAM is 1333 MHz DDR3 of 16 GB. Average time costs of the proposed method, Zhen’s method and Reju’s method are 1.79 s, 14.22 s and 0.17 s, respectively. The main difference between these three methods is the process of SSPs identification, which is the main cause for a significant difference in time cost. Reju’s method can identify SSPs according to single SSP, and only TF vectors in some frequency bins with a larger variance are selected for SSPs identification, therefore, time cost of Reju’s method is the least. SSPs must be identified between two TF vectors in Zhen’s method and the proposed method, which means more time consumption. However, SSPs are also identified in some frequency bins with a larger variance in the proposed method and they can be directly identified by searching the identical normalized TF vectors, instead of finding the sparsest coefficients. Therefore, the time cost of the proposed method is shorter than that of Zhen’s method.

Table 3 shows the average results of source contributions quantitative estimation using different methods, including also the real contributions. It can be clearly seen that source contributions of the proposed method are closer to the real source contributions than those of Zhen’s method and Reju’s method. The average absolute errors of source contributions are also calculated and listed in Table 4. As revealed by the data in Table 4, most of the average contribution errors of the proposed method are the smallest among these three methods, implying that the proposed method has higher accuracy in source contribution. All contribution errors of the proposed method are less than 1.80%, however, three contribution errors are larger than 10% in Zhen’s method and three contribution errors are larger than 4% in Reju’s method. Actually, the accurate source estimation is the premise for correct contribution estimation. Therefore, it can be concluded that the proposed method performs more effectively in recovering source signals and quantitatively estimating source contributions.

## 5. Experimental Study with Cylindrical Structure

Some practical mechanical systems or their partial sections have the shape of cylindrical shells, such as the underwater vehicles. Generally, the sound radiation of underwater vehicles strongly interferes with their performance and safety. Therefore, it is quite important for underwater vehicles to reduce their radiation noise to accomplish tasks successfully. Before that, it is necessary to estimate sound sources in advance. When the number of sensors is smaller than that of sources, UBSS is an excellent method to estimate sources in these cases. Therefore, a test bed with a cylindrical shell structure is used to examine the effectiveness of the proposed method.

**Figure 8:**
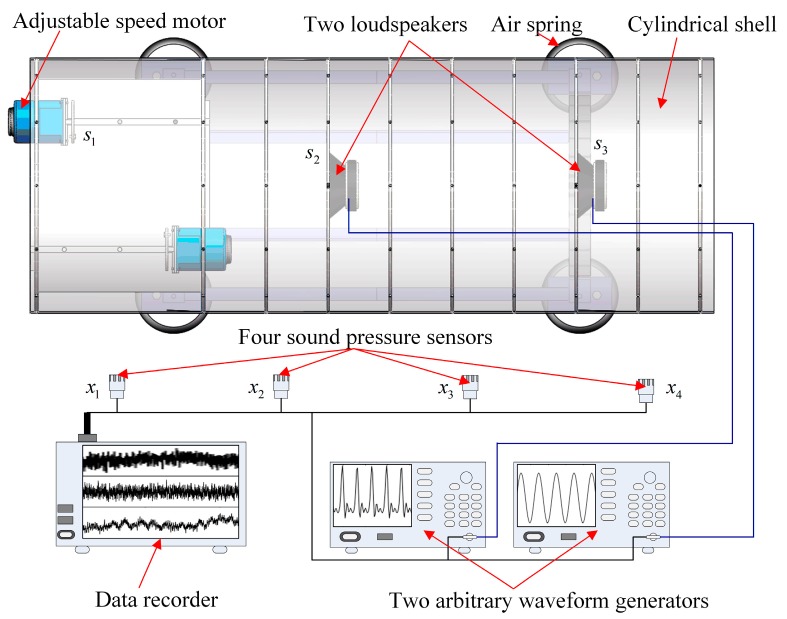
Schematic diagram of the test site.

**Figure 9:**
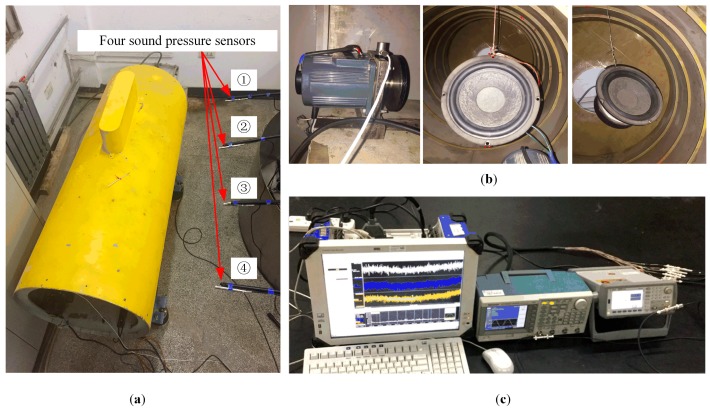
Photos of the test site: (**a**) test bed with a cylindrical shell structure; (**b**) three sources: A motor, and two loudspeakers; (**c**) data recorder and two arbitrary waveform generators.

In the experiments, an adjustable speed motor is used as a vibration source and an eccentric mass disc is driven by the motor to simulate the unbalanced vibration. Two loudspeakers are also used to simulate two radiated noise sources and two arbitrary waveform generators are used to produce two different source signals which are the inputs of these two loudspeakers, respectively. Besides, mixed signals are collected by four sound pressure sensors and are recorded by GEN2i high-speed data recorder. Schematic diagram and photos of the test site are displayed in Figure 8 and Figure 9, respectively.

**Figure 10:**
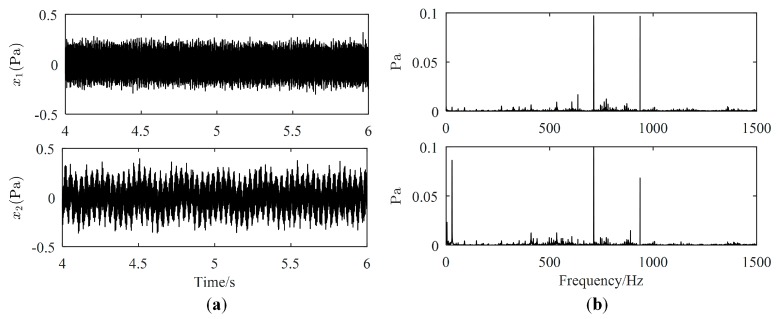
Mixed signals: (**a**) Waveforms; (**b**) Fourier spectrums.

The motor is running at 1740 r/m. Inputs of two loudspeakers, denoted by loudspeaker 1 and loudspeaker 2, respectively, are sine waves of 713 Hz and 917 Hz, respectively. The sampling length and the sampling frequency are 10 s and 5000 Hz, respectively. The second and the fourth mixed signals are selected to estimate three source signals and only a section of data from 4 s to 6 s is used. Waveforms and Fourier spectrums of mixed signals are displayed in Figure 10. From Figure 10a, mixed signals are the superposition of source signals, therefore, we cannot directly obtain waveforms of source signals from mixed signals. From Figure 10b, the major frequencies of source signals can be found in each Fourier spectrum of mixed signals. Therefore, mixed signals need to be further processed to obtain source signals.

**Figure 11:**
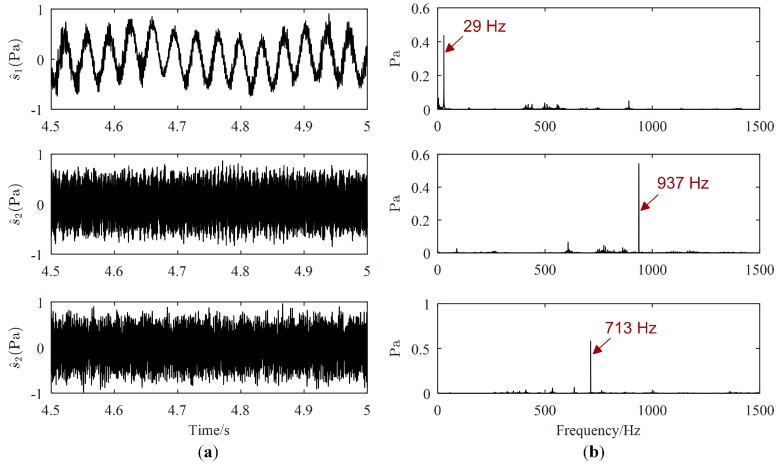
Estimated source signals by the proposed method: (**a**) Waveforms; (**b**) Fourier spectrums.

**Figure 12:**
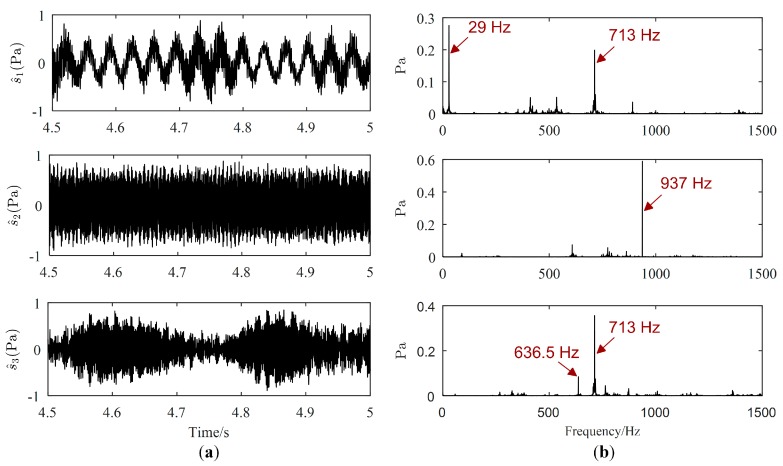
Estimated source signals by Zhen’s method: (**a**) Waveforms; (**b**) Fourier spectrums.

**Figure 13:**
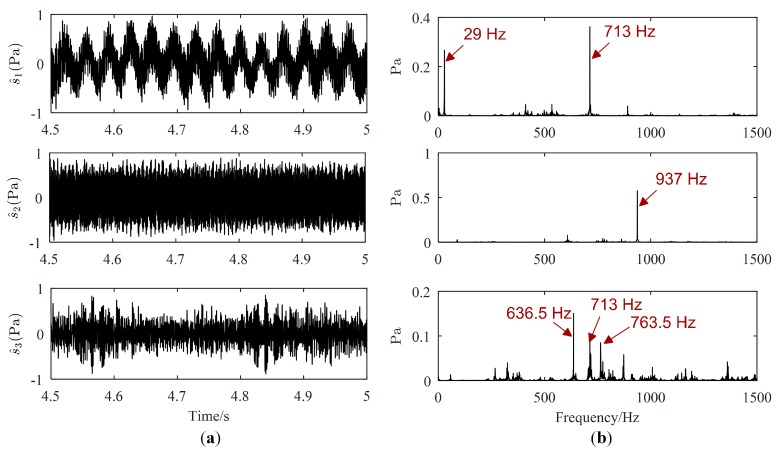
Estimated source signals by Reju’s method: (**a**) Waveforms; (**b**) Fourier spectrums.

Source signals (displayed only from 4.5 s to 5 s) estimated by the proposed method, Zhen’s method and Reju’s method are illustrated in Figure 11, Figure 12 and Figure 13, respectively. As revealed in Figure 11b, the major frequencies of source signals estimated by the proposed method are 29 Hz, 937 Hz and 713 Hz, respectively, which are consistent with the frequencies set in the experiment. However, from Figure 12, both the major frequency 29 Hz of the motor and the major frequency 713 Hz of the loudspeaker 1 is mis-estimated in the same signal, as shown in the Fourier spectrum of the first separated signal. Therefore, 29 Hz and 713 Hz will be mistaken for coming from the same source using Zhen’s method. The major frequencies of the first signal estimated by Reju’s method are also 29 Hz and 713 Hz, as shown in Figure 13. The results tend to illustrate that source signals have been well estimated by the proposed method.

Reju’s method can identify SSP based on the character of single SSP, and the performance of this method will be degraded in noisy cases. In Zhen’s method, to increase the computational efficiency, SSPs are identified only in some TF vectors that are randomly selected from TF vectors of mixed signals. If no or very few SSPs corresponding to a source are included in selected TF vectors, this source will be estimated with large error. And this may be the main reason why the performance of Zhen’s method is not so good as that of the proposed method.

After obtaining the estimated source signals, their contributions to the mixed signals can be calculated and are presented in Table 5. Real source contributions also need to be obtained by the experiment [1]. When one source is stopped, the decreased amount in vibration energy of mixed signals is observed by the sensors. The decreased amount is regarded as the source contribution of the stopped source. The real source contributions are also given in Table 5. We can find the source contributions of the proposed method are closer to the real source contributions than those of Zhen’s method and Reju’s method.

The absolute errors of source contributions are calculated and listed in Table 6. All contribution errors of the proposed method are smaller than those of Zhen’s method and Reju’s method, implying that the proposed method has higher accuracy in source contribution. The largest contribution error of the proposed method is only 6.44%, however, three contribution errors are larger than 12% in Zhen’s method and four contribution errors are larger than 15% in Reju’s method. Actually, accurate estimation of source signals is the precondition for accurate estimation of computational complexity. To some extent, estimation accuracy of contribution increases with the increase in source signal estimation accuracy. As shown in Figure 12 and Figure 13, the first separated signals of Zhen’s method and Reju’s method contains major frequencies of two sources (motor and loudspeaker one). Therefore, the contributions of their first separated signal contain contributions of two real sources, which will lead to an increase in their source contributions. Besides, since part of the contributions of loudspeaker 1 is mis-assigned to their first separated signals, the contribution of their third separated signal will be smaller than the real contributions. Based on a more accurate estimation of source signals, the contribution errors of the proposed method are smaller than those of contrast methods.

Running time is also used to evaluate the efficiency of the methods. Time costs of the proposed method, Zhen’s method and Reju’s method are 1.86 s, 6.42 s and 0.32 s, respectively. Reju’s method could identify SSPs by the property of single SSP, which can have higher efficiency than Zhen’s method and the proposed method. The proposed method can identify SSPs only at some optimal frequency bins and SSPs are identified by directly searching the identical TF vectors in the selected frequency bins, which could the reason why the efficiency of the proposed method is higher than that of Zhen’s method.

It should be noted that the proposed method is designed for off-line processing system because it needs some data to identify SSPs. However, for a real-time monitoring system, we can process the data in a piecewise way by the proposed method. From the experiment, the running time of the proposed method is only 1.86 s to process the data with a length of 2 s. Therefore, we can split the data into a fixed length segment and analyze them by the proposed method.

After source signals are well recovered and source contributions are calculated, the influences of sources on mixed signals can be determined. The vibration sources estimated by the proposed method can be used to machinery condition monitoring and fault diagnosis when source signals are difficult to be directly obtained. The main vibration sources can also be determined according to their contributions. Therefore, some measures can be taken to reduce the impact of the main vibration sources.

## 6. Conclusions

To identify the major vibration and noise sources of the mechanical systems, a novel source contribution quantitative estimation method is proposed for UBSS. The accuracy of the source contribution results relies largely on the accuracy of source recovery. Only by recovering source signals more accurately can we obtain higher accuracy of source contribution estimation. From the results of numerical case studies, the proposed method can not only estimate source signals from their mixtures in underdetermined cases, but also quantitatively estimate the source contributions with average deviations <2%. The results of experimental studies with a cylindrical structure also show the effectiveness of the proposed method in sources restoration and quantitative contribution estimation. The comparative results tend to validate that the proposed method performs more powerfully. Therefore, our method could serve as an effective and promising tool to investigate the major vibration and noise sources, which can benefit vibration and noise monitoring and control of mechanical systems.

## Figures and Tables

**Table 1:** Comparison of average SNRs of estimated mixing matrix.

Methods	SNR (dB)				Average SNR of All Columns
	$a_{1}$	$a_{2}$	$a_{3}$	$a_{4}$	
Zhen’s method	10.01	16.50	20.05	25.92	18.12
Reju’s method	39.63	38.47	25.35	26.15	32.40
The proposed method	43.65	41.82	37.44	39.69	40.65

**Table 2:** Comparison of average SNRs of estimated source signals.

Methods	SNR (dB)				Average SNR of All Sources
	${\hat{s}}_{1}(t)$	${\hat{s}}_{2}(t)$	${\hat{s}}_{3}(t)$	${\hat{s}}_{4}(t)$	
Zhen’s method	9.61	9.65	7.39	6.99	8.41
Reju’s method	10.18	9.94	8.00	8.57	9.17
The proposed method	12.56	12.40	9.78	11.93	11.66

**Table 3:** Average contribution comparison of estimated source signals.

Mixed Signals	Methods	Contributions (%)			
		$s_{1}(t)$	$s_{2}(t)$	$s_{3}(t)$	$s_{4}(t)$
$x_{1}\left(t\right)$	Zhen’s method	0.2015	0.2790	0.1227	0.1049
	Reju’s method	0.2180	0.2790	0.1544	0.2379
	The proposed method	0.2368	0.2857	0.1734	0.3090
	Real contributions	0.2314	0.2780	0.1630	0.3018
$x_{2}\left(t\right)$	Zhen’s method	0.2152	0.2799	0.1534	0.1083
	Reju’s method	0.2591	0.2877	0.1665	0.2090
	The proposed method	0.2615	0.2963	0.1998	0.2517
	Real contributions	0.2529	0.2872	0.1862	0.2447
$x_{3}\left(t\right)$	Zhen’s method	0.3791	0.2791	0.1400	0.2591
	Reju’s method	0.4275	0.2810	0.0854	0.1239
	The proposed method	0.4381	0.3079	0.1009	0.1594
	Real contributions	0.4229	0.2939	0.0906	0.1501

**Table 4:** Comparison of average contribution errors of estimated source signals.

Mixed Signals	Methods	Contribution Errors (%)			
		$s_{1}(t)$	$s_{2}(t)$	$s_{3}(t)$	$s_{4}(t)$
$x_{1}\left(t\right)$	Zhen’s method	6.52	2.22	6.57	21.45
	Reju’s method	1.70	1.28	3.89	6.72
	The proposed method	**0.84**	**0.78**	**1.13**	**1.37**
$x_{2}\left(t\right)$	Zhen’s method	6.00	2.71	6.10	16.32
	Reju’s method	1.26	**0.92**	4.47	4.09
	The proposed method	**1.05**	1.00	**1.37**	**1.15**
$x_{3}\left(t\right)$	Zhen’s method	6.14	3.26	5.84	13.51
	Reju’s method	1.11	2.25	2.03	3.08
	The proposed method	**1.80**	**1.78**	**1.03**	**1.04**

**Table 5:** Contribution comparison of estimated source signals.

Mixed Signals	Methods	Contributions (%)		
		$s_{1}(t)$	$s_{2}(t)$	$s_{3}(t)$
$x_{1}\left(t\right)$	Zhen’s method	21.82	46.50	39.00
	Reju’s method	28.29	43.30	12.98
	The proposed method	3.10	47.43	50.27
	Real contributions	7.31	47.15	47.64
$x_{2}\left(t\right)$	Zhen’s method	59.90	17.18	24.36
	Reju’s method	61.91	16.56	2.19
	The proposed method	38.97	16.96	40.20
	Real contributions	45.41	17.09	36.54

**Table 6:** Comparison of contribution errors of estimated source signals.

Mixed Signals	Methods	Contribution Errors (%)		
		$s_{1}(t)$	$s_{2}(t)$	$s_{3}(t)$
$x_{1}\left(t\right)$	Zhen’s method	14.51	0.65	8.64
	Reju’s method	20.98	3.85	34.66
	The proposed method	**4.21**	**0.28**	**2.63**
$x_{2}\left(t\right)$	Zhen’s method	14.49	0.09	12.18
	Reju’s method	16.50	0.53	34.35
	The proposed method	**6.44**	**0.13**	**3.66**
